# Effect of Winter Outdoor Physical Activity on Body Composition and Motor Performance of Polish Adult Men

**DOI:** 10.3390/healthcare11162348

**Published:** 2023-08-20

**Authors:** Monika Stanaszek, Jarosław Fugiel, Sławomir Kozieł, Anna Sebastjan, Agnieszka Suder, Zofia Ignasiak

**Affiliations:** 1 Department of Physiotherapy, Witelon Collegium State University, 59-220 Legnica, Poland; 2 Department of Biostructure, Wroclaw University of Health and Sport Sciences, 51-612 Wroclaw, Poland; 3 Department of Anthropology, Hirszfeld Institute of Immunology and Experimental Therapy, Polish Academy of Sciences, 53-114 Wroclaw, Poland; 4 Department of Anatomy, University of Physical Education, 31-571 Cracow, Poland

**Keywords:** body mass index, outdoor physical activity, body composition, motor fitness

## Abstract

There is a relationship between physical activity and environmental factors, including weather conditions. Winter should not be a season in which physical activity is abandoned. Previous studies indicate that reducing the daily level of physical activity in adults has a negative impact on their motor proficiency and respiratory endurance, which subsequently translates into diminished quality of life. The aim of the study was to assess the impact of winter outdoor physical activity on somatic parameters, body composition, and motor performance of adult men (age 45.4 ± 5.9 years) involved in regular physical activity (physically active PA, *n* = 31) during the winter season (study group) compared to the control group of physically inactive men (PI, *n* = 22). Somatic parameters and selected parameters of motor fitness (Eurofit for Adults) were measured in both groups twice, at the beginning (pre) and at the end (post) of the winter season. An analysis of variance (ANOVA) with repeated measures and a post hoc LSD test were performed to evaluate the difference between the mean parameter values. In the PA group, a decrease in body fat mass, waist circumference, and abdominal obesity indicators, as well as an increase in skeletal muscle mass were confirmed. Improvements in motor performance parameters, i.e., flexibility, the speed of upper limb movements, hand and forearm muscle grip strength, the strength of the lower part of the torso, and cardiorespiratory endurance were also observed. Regular physical activity in the open air during the winter brings measurable health benefits, positively influencing the body composition and motor efficiency of adult men.

## 1. Introduction

According to a Eurobarometer study (investigation of public opinions of the European Union member states, commissioned by the European Parliament), Poles belong to the group of the 11 least physically active residents of the European Union [1]. The research shows that over 56% of the Polish population does not participate in any sports activities at all. The result falls below the European Union average, which is 43%. The leaders in the engagement of sporting activities were the residents of Scandinavian countries (Swedes, Finns, and Danes), along with the residents of the Netherlands, where an average of 84% of adult citizens participate in sports during their leisure time. According to the Central Statistical Office data [2], the percentage of adult Poles engaging in sports or recreational exercises decreases with age. Among people aged 30–39, 28.5% were active, while in the 40–49 age group, it was 19.3%. In the 50–59 age group, only 13.5% of people engaged in any form of physical activity. 

The reduction in daily physical activity levels among adult individuals invariably exerts a negative impact on their motor proficiency and respiratory endurance, which subsequently translates into diminished quality of life [3]. The consequences of a lack of physical activity are negative health effects, faster aging of the body, and an increased risk of many civilization diseases [4,5,6]. Sports participation is closely linked to socioeconomic status (SES). People with higher levels of education who assess their financial situation well are decidedly more active. A higher level of education is associated with greater knowledge about health and ways of taking care of it. It often motivates people to make the decision to work regularly on physical fitness [7]. 

It is increasingly believed that physical activity is related to external environmental conditions and has a seasonal pattern [8]. The level of physical activity may also differ depending on the time of year [9]. Ambient temperature, sunlight, wind speed, and precipitation can predispose or hinder physical activity practiced in an open area [9,10,11,12]. Numerous studies indicate that people are more active in warmer seasons compared to colder ones. There is a significant decrease in activity with decreasing air temperature and, above all, with increasing precipitation (rain or snow). Additionally, it has been shown that the length of the day influences the time spent on physical activity [10,11,13].

Winter should not be a season in which people give up physical activity. It was proven that outdoor activity during the winter period results in burning more energy than at other times of the year. This is due to a need for additional energy expenditure to heat the body. Furthermore, physical activity at this time of year offers more benefits than in the summer: it increases the production of immune bodies; allows more oxygen to reach the brain, thereby improving its function; and increases the number of endorphins that protect against the winter mood slump [8,13]. Typically, physical activity during the winter is significantly lower. Such situations can lead to unfavorable changes in morphological and motor fitness. Engaging in regular physical activity from autumn to spring provides an opportunity to maintain motor fitness independently of the seasonal rhythm. 

It is known that there are serious problems with obtaining anthropometric measurement data and conducting motor tests among the adult working population. It is particularly difficult to perform measurements on a group of men who are reluctant to participate in this type of research. They often cite a lack of time as the reason. Given the above, the research was conducted among physically active men aged 40–60, whose physical activity profile was focused on locomotion in open spaces (cross-country skiing, downhill skiing, skitouring, cross-country running, and cycling).

The aim of the study was to evaluate the effect of winter outdoor physical activity on somatic parameters, body composition, and motor fitness of adult men engaging in systematic physical activity during the winter compared to a control group leading a sedentary lifestyle.

## 2. Materials and Methods

### 2.1. Materials

The research project was approved by the Ethics Committee for Scientific Research at the University of Physical Education in Wrocław, Poland (no. 24/2017). 

The participants in the study were adult men living in the Jelenia Góra Valley, located in the Polish Sudety Mountains. The bottom of the Jelenia Góra Valley is situated at an altitude of 350–420 m above sea level, and the surrounding mountain ranges reach significant absolute heights. The highest mountain range surrounding the Jelenia Góra Valley, as well as the highest range in the Sudety, is the Karkonosze Mountains with the peak of Śnieżka (1602 m above sea level). The climate in this orographic unit is characterized as mountainous and submountainous. The average annual temperature ranges from 7.6 °C to 0.4 °C. There is a high average amount of rainfall, ranging from 750 mm to as much as 1450 mm. 

The access to recreational and sports facilities that enables physical activity in open spaces was similar for all the men. The participants in the project were in good health, confirmed by written consent from their physicians to participate in the study, and no contraindications to increased physical activity and the planned scope of the study. Each participant was informed of the purpose of the study, the type and manner of conducting it, and the possibility of resigning from the study without giving a reason. 

The study inclusion criteria were male sex, age between 40 and 60, written consent to take part in the examinations, and a lack of contraindications for undertaking physical activity.

The exclusion criteria were a lack of consent to participate in examinations and motion limitations that made practicing outdoor sports impossible.

In order to obtain information on the frequency of physical activity in leisure time during the winter season, the questionnaire method was used. The subjects were asked about the frequency (how many times a week), the average duration of a single effort (in minutes or hours), and the form of activity in outdoor sports (for example: cross-country skiing, downhill skiing, skitouring, cross-country running, and cycling). Two groups were selected:

1. A group of men with high physical activity (physically active PA), *n* = 31, 43.2 ± 5.8 years. These men demonstrated a strictly defined, systematic (3–5 times a week) physical activity that they maintained from December to April. The participants engaged in outdoor sports such as cross-country skiing, downhill skiing, skitouring (*n* = 13, 41.9%), cross-country running (*n* = 14, 45.2%), and cycling (*n* = 4, 12.9%). The frequency of physical activity ranged from a minimum of 3 to a maximum of 5 days per week. The duration of individual physical activity ranged from 1 to 1.5 h.
2. A control group of men with low physical activity (physically inactive PI), *n* = 22, 47.5 ± 6.0 years. These men led a sedentary lifestyle from December to April.

The education structure in both groups of men showed no significant differences between the groups (χ^2^ = 0.06; *p* > 0.05). Higher education (bachelor’s degree and above, 64.2%) predominated in both groups. Lower education (secondary and lower) was declared by 35.8% of the respondents in both groups.

Due to the difficulty in recruiting participants willing to take part in the study, it was conducted over two winter seasons from December 2017 to April 2019. In the first winter season of 2017/2018, 35 men started the study, and 31 completed it. In the next season of 2018/2019, 30 men started the study, and 22 completed it. During this period (December to April), the same group of men was tested twice. 

### 2.2. Methods

The following measurements were taken twice, at the beginning (pre) of the winter season and at the end (post):

#### 2.2.1. Somatic Measurements

Body height (Ht) [cm] and body mass (BM) [kg] were measured with an accuracy of 0.1 cm and 0.1 kg using a SECA model 764 device. Waist and hip circumferences [cm] were measured with a tape measure with an accuracy of 0.5 cm. Based on the obtained measurements, the following indicators were calculated:

- Body Mass Index (BMI) [kg/m^2^]
- Waist-to-hip ratio (WHR), an indicator of fat distribution
- Waist-to-height ratio (WHtR), an indicator of fat distribution relative to body height.
  WHtR values ≥ 0.50 indicate an increased risk of cardiovascular disease and diabetes.

#### 2.2.2. Body Composition

The In-Body 230 body composition analyzer (InBody Co., Ltd. in Seoul, Republic of Korea) was used to assess body tissue composition. The measurements were performed according to the manufacturer’s recommendations. The analyzed parameters were:

- SMM—Skeletal Muscle Mass [%]
- BFM—Body Fat Mass [%]

#### 2.2.3. Motor Fitness Test

Selected tests from the European Physical Fitness Test—Eurofit for Adults were used to assess motor fitness. The tests were performed in the order recommended by the authors [14]:

- Sit-and-reach test [cm]—assesses flexibility (range of motion of the spine and hip joints)
- Plate tapping test [s]—assesses the speed of upper limb movements
- Hand grip test [kg]—assesses hand and forearm muscle grip strength (static strength assessment)
- Dynamic sit-up test [*n*]—assesses the strength of the lower part of the torso
- Endurance shuttle run test [*n*]—assesses cardiorespiratory endurance.

### 2.3. Statistical Analysis

Descriptive statistical methods were used for the data analysis. To determine the probability of data having a normal distribution, the Shapiro–Wilk test was used. Parametric tests were used after accepting the hypothesis of normal distribution. Basic statistical characteristics, such as arithmetic mean (M), standard deviation (SD), and minimum (Min) and maximum (Max) values of a given parameter, were used. 

An analysis of variance (ANOVA) with repeated measures and a post hoc LSD (Least Significant Difference) test was used to assess the difference between the mean values of the parameters in the first and second evaluations of the groups. 

The Spearman rank correlation was used to determine the associations between variables. Percentage and numerical comparisons were used to describe the study groups in terms of selected socio-economic factors. 

The level of statistical significance was assumed for a *p*-value of ≤0.05. The Statistica 13 TIBCO Software Inc. (Palo Alto, CA, USA, 2017) data analysis software system, version 13, was used for calculations.

## 3. Results

When physically active and inactive men participated in the study, they were characterized by similar average height and weight values (Table 1). After the winter period, the body weight of physically active men decreased by 0.67 kg, and the change was statistically significant. The physically inactive group showed a slight increase in body weight.

The average values of waist and hip circumferences before and after the evaluation in the physically active group were lower than those of the inactive men in the control group. After the winter period, these values in physically active men decreased, showing statistical significance. The same characteristics in the control group of men increased in the post-winter test (Table 1 and Table 2).

The average BMI value in the physically active group in the pre-test slightly exceeded the expected value. In the post-winter test, the BMI value in this group decreased to the normal category (between 18.5 and 24.99), and this change was statistically significant. The physically inactive group had higher mean BMI values in both tests, falling into the overweight category. The difference between the groups in the post-test was significant (Table 1 and Table 2).

Measurements of the WHR index in the first and second evaluations did not show changes in its value in the physically active group of men. For maintaining health, a desired result of up to 0.9 is recommended, which is considered normal. The mean score for the active group was 0.91, placing it on the border of gynoid (hip-thigh) and android (abdominal) obesity. The control group was characterized by abdominal obesity, and the winter period resulted in a significant increase in the value of this index. Both in the first and second evaluations, the differences in the values of the WHR index between the groups were significant (Table 1 and Table 2).

The mean values of the WHtR index in the physically active group of men in the first and second evaluations were similar: 0.50% and 0.49%. Both values were within the normal range. The control group exceeded the expected values in both evaluations. The mean value of the index after the winter period was higher than before. The differences between the two groups of men were significant (Table 1 and Table 2).

In the pre-winter test, the group of physically active men had a higher percentage of skeletal muscle mass (SMM%) in total body mass compared to the control group. After the winter period, the physically active men increased their percentage of SMM. The percentage of skeletal muscle mass in the group of physically inactive men decreased. The differences between the groups in this parameter in the first and second evaluations were significant. It should be emphasized that these differences increased in favor of the physically active group (Table 1 and Table 2).

In the first evaluation, the group of physically active men had an average value of 16.8% of body fat in total body mass. The winter period of activity resulted in a significant reduction of 1.4% in the percentage of body fat in this group. The group of inactive men maintained the percentage of body fat at a constant level of 21.8–21.9%. The differences between the groups regarding this characteristic were considerable. In the first evaluation, the tested group (PA) had a significantly lower percentage of body fat compared to the control group. In the second evaluation, the differences increased in favor of the physically active men. The differences between the groups in the first and second tests were significant (Table 1 and Table 2).

In all motor fitness tests conducted, the physically active group of men achieved better results than the control group. The differences between the groups increased in the second evaluation (Table 3), and all analyzed tests were statistically significant (Table 4).

In the test evaluating flexibility, the PA group achieved better average results than the control group in both sessions. The differences were significant in the second evaluation, conducted after the winter period. The range of motion of the spine and hip joints in the physically active group of men improved in the second evaluation, while in the control group, it decreased. Both changes were statistically significant (Table 3 and Table 4).

The time needed to complete the speed test of movements of the upper limb was shorter in the physically active group compared to the inactive group in both tests. Physically active men significantly reduced the time to perform the test in the second evaluation (Table 3 and Table 4).

Significantly higher grip strength was observed in the physically active group in both evaluations. The physically active group of men achieved a statistically better result in the second test compared to the first one. The physically inactive group achieved a statistically worse result in the same comparison (Table 3 and Table 4).

In both evaluations, the physically active group obtained significantly better results in lower torso muscle strength than men in the control group. In the second evaluation, the differences between the groups deepened in favor of the physically active group, where the results of the second evaluation were significantly higher than in the first one (Table 3 and Table 4).

In the test evaluating cardiorespiratory endurance, the PA group achieved significantly better results in both evaluations compared to the control group. In the post-test, the results of the PA group significantly improved, while in the control group, their results significantly decreased (Table 3 and Table 4).

## 4. Discussion

The obtained results unambiguously indicate a significant change in somatic parameters, as well as an improvement in motor fitness parameters, in a group of men engaging in systematic physical activity (3–5 times a week) during the winter season. In particular, a significant reduction in overall body fat mass and a decrease in abdominal distribution in physically active men should be noted. Similarly, all motor fitness parameters of physically active men improved in the winter season compared to the control group.

Currently, increased body weight is considered a common problem among people of all ages. Preventing overweight and obesity is crucial because it reduces the risk of civilization diseases. Scientists have demonstrated a high correlation between the time devoted to physical activity and the occurrence of obesity [15]. Westerterp [16], monitoring seasonal changes in body weight and composition depending on physical activity, indicated that body weight shows clear seasonal variability related to changes in physical activity. The body weight of the subjects reached its highest level in the cold winter months when physical activity levels were lowest. Arnardottir et al. [17] demonstrated significant changes in somatic parameters after a year of monitoring the physical activity of adult residents of Reykjavik. The study was conducted using the ActiGraph GT3X accelerometer, which measured physical activity in summer and winter. In winter months, characterized mainly by very low sunlight, lower physical activity was observed compared to summer months when it is bright almost around the clock. The differences in results were statistically significant, which was reflected in the BMI value. The authors noted a negative correlation between physical activity and BMI and a positive correlation between a sedentary lifestyle and the same indicator.

In this study, significant differences in the values of basic somatic parameters were observed among the subjects. Body weight and BMI were significantly different in both the active and control groups. In the group of men not engaging in physical activity during the winter months (December–April), increasing overweight was observed. Physically active men reduced body weight, and the BMI value shifted within the norm. Similar results were obtained by Burtscher et al. [18], examining a group of Austrian adults who regularly engaged in recreational skiing. The authors demonstrated that men who ski recreationally have a lower BMI than the control group. Favorable changes in body composition were demonstrated by Niederseer et al. [19], subjecting a randomly selected group to ski health training. The training lasted for 12 weeks and was conducted by a licensed ski instructor. After the training cycle, a statistically significant reduction in body fat mass was observed in the study group. Müller et al. [20] also demonstrated the positive impact of physical activity on lung capacity and explosive strength during the winter season.

The results of research on the adult population of England conducted by Clemes et al. [21] seem to be interesting. The authors showed that adults with a BMI within the normal range were more active during the winter compared to individuals whose BMI was on the border of overweight and obesity. Seasonal differences in physical activity levels can have a significant impact on reducing immunity and increasing the incidence of cardiovascular diseases during the winter when physical activity among adults decreases significantly. This was hypothesized by Cepeda et al. [8] based on research conducted on a group of adult residents of Rotterdam and Klompsta et al. [9] studying Swedish adults.

Engaging in physical activity during the winter period among active men had a positive effect on the distribution of WHR and WHtR adipose tissue. In contrast, inactive men experienced a deepening of abdominal obesity, which is associated with a higher degree of cardiovascular risk [22].

Regular physical activity during the winter period led to an improvement or maintenance of motor fitness. The most visible improvement was noted in the range of static strength, lower torso muscle strength, and cardiorespiratory endurance. Despite the presented health benefits of physical activity during the winter period, a large percentage of adults opt out of being active during this season. Research conducted by Li et al. [5] in Toronto showed that older adults do not engage in outdoor activities due to fear of slippery surfaces and the risk of falling. During the period of snow cover, temperature drop, and precipitation, the most commonly undertaken activity is walking [23]. The health effects of this type of activity may be insufficient and may not contribute to the maintenance or improvement of health conditions.

The results of our own research speak to the necessity of incorporating regular exercise into the population of adult individuals leading predominantly sedentary lifestyles. Engaging in physical activity outdoors was associated with difficulties such as adverse weather conditions (wind, precipitation, low daily temperatures, and short sunshine duration). All study participants were employed, which undoubtedly limited regular health training.

It is particularly important for ageing adults to maintain regular physical activity, as this allows them to avoid premature health problems and, as a result, maintain their work efficiency. Engaging in regular physical activity resulting from the individual body’s capabilities in adulthood provides a chance to enter old age with a higher health potential. Additionally, the unavoidable involutional processes in physically active individuals occur at a different (slower) pace [24].

Despite obtaining unambiguous results indicating the beneficial effects of physical activity, it should be mentioned that our study had certain limitations. Firstly, the men belonging to the active group in the initial study showed significant differences in several somatic parameters, determining the type of fat distribution and its size. Similarly, significant differences were demonstrated in motor fitness parameters, especially in the shuttle run endurance test. Secondly, both groups showed significant differences in age at the beginning of the study (t = 2.6; *p* ≤ 0.05). The fact that the active group of men was over 4 years older suggests a significantly greater awareness and care for health. Nevertheless, this fact somewhat disturbs the randomness of the selection of individual groups. The obtained results are specific for the Polish population taking into account the aspects of winter weather conditions.

## 5. Conclusions

In summary, engaging in regular physical activity in open areas during the winter season brings measurable health benefits. It has a positive effect on somatic parameters and body composition, allowing for their maintenance or favorable health-related changes. The level of motor fitness in the men who regularly participated in sports in open spaces during the winter season was significantly higher than the level of motor fitness in men who passively spend their free time, and the differences significantly increased in favor of the men who engaged in systematic physical activity after this period. At the same time, the level of motor fitness in the control group of men decreased. Hence, the conclusion is that there is a need for action on the part of decision-making centers (local governments or non-governmental organizations) and media (including social media) to raise awareness among adults about the necessity of also engaging in physical activity during the winter season.

## Figures and Tables

**Table 1:** Statistical characteristics of selected features and somatic indicators of active men (PA) and physically inactive men (PI) in the pre- and post-winter tests.

Variables	Physically Active Group (PA) *n* = 31								Physically Inactive Group (PI) *n* = 22							
	Pre				Post				Pre				Post			
	M	SD	Min	Max	M	SD	Min	Max	M	SD	Min	Max	M	SD	Min	Max
Height [cm]	179.4	5.6	168.0	192.0	-	-	-	-	178.6	6.6	166.0	190.0	-	-	-	-
BM [kg]	80.8	7.5	66.0	102.0	80.1	7.5	65.9	101.2	84.2	11.3	65.0	109.5	84.9	11.2	66.0	108.3
WC [cm]	88.9	6.1	76.5	103.0	88.6	5.9	75.5	103.0	95.7	6.1	84.0	109.0	96.5	6.5	84.0	111.0
HP [cm]	98.0	4.3	86.0	104.0	97.8	4.4	84.0	104.0	100.6	4.5	92.0	112.0	100.7	4.4	92.5	112.0
BMI [kg/m^2^]	25.09	2.06	21.40	29.71	24.89	2.06	21.33	30.17	26.34	2.60	23.15	32.70	26.55	2.52	23.25	32.34
WHR	0.91	0.05	0.82	1.05	0.91	0.05	0.82	1.05	0.95	0.03	0.88	1.02	0.96	0.04	0.88	1.04
WHtR [%]	0.50	0.03	0.45	0.59	0.49	0.03	0.45	0.59	0.54	0.03	0.48	0.59	0.54	0.03	0.48	0.61
SMM [%]	47.6	2.50	41.5	51.4	48.2	2.33	43.4	52.1	44.6	2.6	39.9	48.3	44.3	2.1	41.1	47.6
BFM [%]	16.8	4.00	15.7	10.8	15.4	3.8	9.5	22.8	21.9	4.3	15.1	29.1	21.8	3.5	16.3	27.6

M—mean, SD—standard deviation, Min—minimum, Max—maximum, BM—Body Mass, WC—Waist Circumference, HP—Hip Circumference, BMI—Body Mass Index, WHR—Waist to Hip Ratio, WHtR—Waist to Height Ratio, SMM—Skeletal Muscle Mass, BFM—Body Fat Mass.

**Table 2:** Evaluation of the significance of differences between the mean values of somatic parameters in the group of physically active men and physically inactive men (PA-PI) between pre- and post-winter tests—analysis of variance with repeated measures.

Variables	Main Effects						Probabilities for Post Hoc, Test LSD; *p*-Value			
	Activity Group PA-PI		Pre-Post		Interaction, Activity—Test		Pre-Post		PA-PI	
	F	*p*	F	*p*	F	*p*	PA	PI	Pre	Post
Height [cm]	0.24	0.6261	0.71	0.4048	0.71	0.4048	0.1981	1.0000	0.6294	0.6227
BM [kg]	2.59	0.1136	<0.001	0.9860	8.00	0.0067	0.0338	0.0685	0.1821	0.0694
WC [cm]	18.67	<0.0001	3.93	0.0527	22.56	<0.0001	0.0366	0.0001	0.0002	<0.0001
HP [cm]	4.83	0.0326	1.46	0.2325	7.09	0.0103	0.0041	0.3461	0.0446	0.0237
BMI [kg/m^2^]	5.29	0.0256	0.00	0.9964	8.47	0.0053	0.0280	0.0632	0.0546	0.0119
WHR	14.68	0.0004	5.99	0.0179	11.15	0.0016	0.4921	0.0004	0.0011	0.0001
WHtR [%]	22.13	<0.0001	3.80	0.0569	23.04	<0.001	0.0314	<0.0001	0.0001	<0.0001
SMM [%]	28.56	<0.0001	0.97	0.3299	9.00	0.0042	0.0032	0.1933	<0.0001	<0.0001
BFM [%]	30.07	<0.0001	8.08	0.0064	6.82	0.0118	0.0001	0.8804	<0.0001	<0.0001

*p*—statistically significant value (*p* ≤ 0.05), BM—Body Mass, WC—Waist Circumference, HP—Hip Circumference, BMI—Body Mass Index, WHR—Waist to Hip Ratio, WHtR—Waist to Height Ratio, SMM—Skeletal Muscle Mass, BFM—Body Fat Mass.

**Table 3:** Descriptive statistics of motor fitness parameters of physically active men (PA) and physically inactive men (PI) in the pre- and post-winter tests.

Motor Skills	Physically Active Group (PA) *n* = 31								Physically Inactive Group (PI) *n* = 22							
	Pre				Post				Pre				Post			
	M	SD	Min	Max	M	SD	Min	Max	M	SD	Min	Max	M	SD	Min	Max
sit-and-reach [cm]	30.6	8.6	14.0	45.0	31.8	8.8	14.5	48.0	26.2	7.9	13.5	48.0	25.5	7.3	12.0	45.0
plate tapping [s]	10.4	0.9	9.1	12.4	10.0	0.7	9.1	11.4	11.4	1.0	9.1	13.6	11.3	1.2	9.2	14.0
hand grip [kg]	59.4	8.5	41.0	76.0	60.5	8.7	41.0	80.0	54.5	10.1	33.0	74.0	53.5	9.7	34.0	73.0
dynamic sit-up [*n*]	25.0	3.9	18.0	34.0	27.0	4.2	19.0	35.0	19.0	4.0	11.0	29.0	18.0	4.1	10.0	29.0
endurance shuttle run test [*n*]	77.0	13.6	52.0	103.0	83.0	15.5	52.0	108.0	42.0	14.4	19.0	66.0	39.0	14.4	18.0	66.0

M—mean, SD—standard deviation, Min—minimum, Max—maximum.

**Table 4:** Assessment of the significance of differences between the average values of motor fitness parameters in the group of physically active and physically inactive men (PA-PI) between pre- and post-winter tests—analysis of variance with repeated measures.

Motor Skills	Main Effects						Probabilities for Post Hoc, Test LSD; *p*-Value			
	Activity Group PA-PI		Pre-Post		Interaction, Activity—Test		Pre-Post		PA-PI	
	F	*p*	F	*p*	F	*p*	PA	PI	Pre	Post
sit-and-reach [cm]	5.44	0.024	0.76	0.388	21.25	<0.001	<0.001	0.018	0.062	0.009
plate tapping [s]	21.00	<0.001	6.90	0.011	4.69	0.035	0.001	0.764	<0.001	<0.001
hand grip [kg]	5.57	0.022	0.06	0.816	14.91	<0.001	0.003	0.022	0.059	0.008
dynamic sit-up [*n*]	36.65	<0.001	3.62	0.063	26.50	<0.001	<0.001	0.039	<0.001	<0.001
endurance shuttle run test [*n*]	96.32	<0.001	3.98	0.051	45.76	<0.001	<0.001	0.003	<0.001	<0.001

*p*—statistically significant value (*p* ≤ 0.05).

## Data Availability

Data are available on request from the corresponding author.
